# Lumbar Transverse Process Stress Fractures in Adolescent Athletes: A Report of Two Rare Cases

**DOI:** 10.7759/cureus.95716

**Published:** 2025-10-29

**Authors:** Takao Minami, Motohiro Okada, Yasuhiro Nakane, Munehito Yoshida

**Affiliations:** 1 Orthopaedic Surgery, Sumiya Orthopaedic Hospital, Wakayama, JPN; 2 Orthopaedic Surgery, Sumiya Orhthopaedic Hospital, Wakayama, JPN

**Keywords:** adolescent athletes mr bone imaging, conservative management, lumbar transverse process fracture, mr bone imaging, stress fracture

## Abstract

Low back pain is one of the most frequent musculoskeletal complaints among adolescent athletes. Lumbar transverse process fractures are usually associated with high-energy trauma, such as traffic accidents and falls, and have also been reported in contact and impact sports such as football and skiing. In contrast, stress fractures of the lumbar transverse process without a traumatic episode are rare, with only a few cases described in the literature.

We report two rare cases of lumbar transverse process stress fractures in adolescent athletes. A 16-year-old football player developed progressive low back pain without a history of trauma, and an MRI with magnetic resonance (MR) bone imaging revealed a stress fracture of the L3 transverse process. A 15-year-old ballet dancer presented with buttock pain, and an MRI with MR bone imaging demonstrated a fracture at the tip of the L5 transverse process. Both patients were treated conservatively with activity restriction and rehabilitation. Pain improved at around three weeks, CT at four weeks confirmed union, and return to sport was achieved at approximately eight weeks.

Diagnosis can be challenging, as plain radiographs often fail to detect these fractures, while MRI and MR bone imaging enable early recognition. MRI bone imaging provides CT-like visualization of cortical bone, allowing fracture assessment while minimizing radiation exposure in adolescents. Because delayed diagnosis may lead to nonunion and chronic low back pain, awareness of this entity is essential. Lumbar transverse process stress fractures should be included in the differential diagnosis of low back pain in adolescent athletes, and prompt imaging evaluation facilitates early management and favorable return-to-sport outcomes.

## Introduction

Low back pain is common among adolescent athletes, affecting 10-15% across various sports [1,2], and while lumbar transverse process fractures typically result from high-energy trauma such as traffic accidents or falls, stress fractures without trauma are exceedingly rare [3-8]. This report aims to describe two rare cases of lumbar transverse process stress fractures and discuss their anatomical and biomechanical mechanisms. Anatomically, the lumbar transverse process serves as the attachment site for several muscles and ligaments, including the psoas major, quadratus lumborum, and iliolumbar ligament [9,10]. Repetitive trunk and hip movements can generate substantial traction forces on these structures, which may result in stress injury even in the absence of direct trauma.

Lumbar transverse process stress fractures are difficult to detect on plain radiographs and are easily overlooked; therefore, MRI and CT are required for accurate evaluation and early diagnosis [7,8]. When recognized promptly, conservative management generally yields favorable outcomes, whereas delayed diagnosis may lead to nonunion or chronic low back pain. This report presents two rare cases of lumbar transverse process stress fractures in adolescent athletes without a history of trauma: one involving L3 in a football player and one involving L5 in a classical ballet dancer. The anatomical and biomechanical mechanisms underlying these injuries are discussed. Written informed consent was obtained from both patients.

## Case presentation

Case 1

**Figure 1:**
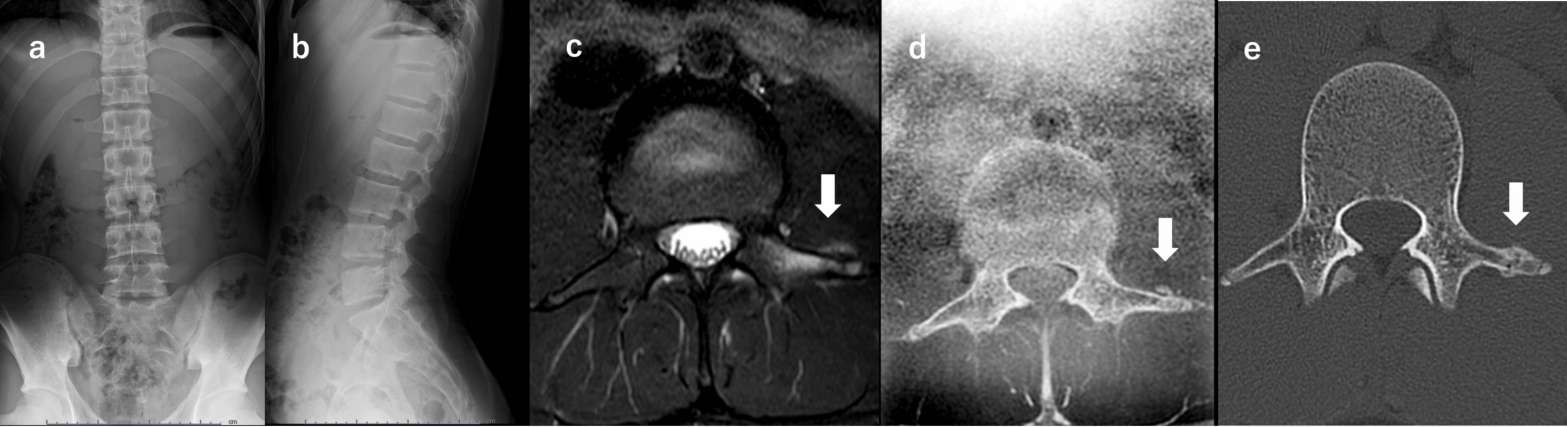
Sequential imaging findings of an L3 transverse process stress fracture in Case 1 (a, b) Plain radiographs showing no abnormalities, (c) Axial Short Tau Inversion Recovery (STIR) MRI demonstrating high signal intensity at the left L3 transverse process and adjacent soft tissue (white arrow), (d) MR bone imaging revealing a fracture line and callus-like change (white arrow), (e) Axial CT image at four weeks confirming bone union (White arrow).

A 16-year-old male high school football player presented with low back pain of two months’ duration. He reported no trauma, and symptoms developed progressively. The pain intensified, leading him to restrict training, but after a temporary improvement, he resumed full activity. Six weeks after onset, symptoms recurred, preventing participation in practice and prompting medical evaluation. Examination revealed left-sided low back pain during trunk flexion, extension, and rotation. The straight leg raise was 60/60 with tightness, without neurological deficits. Plain radiographs were unremarkable. MRI axial Short Tau Inversion Recovery (STIR) sequences demonstrated signal alteration in the left L3 transverse process and adjacent soft tissue. MR bone imaging showed a fracture line and callus-like change. A diagnosis of L3 transverse process stress fracture was made, and conservative management with activity restriction and rehabilitation was instituted. Pain improved after about three weeks, and CT at four weeks confirmed union (Figure 1). Progressive training was resumed, and return to play was achieved at eight weeks.

Case 2

**Figure 2:**
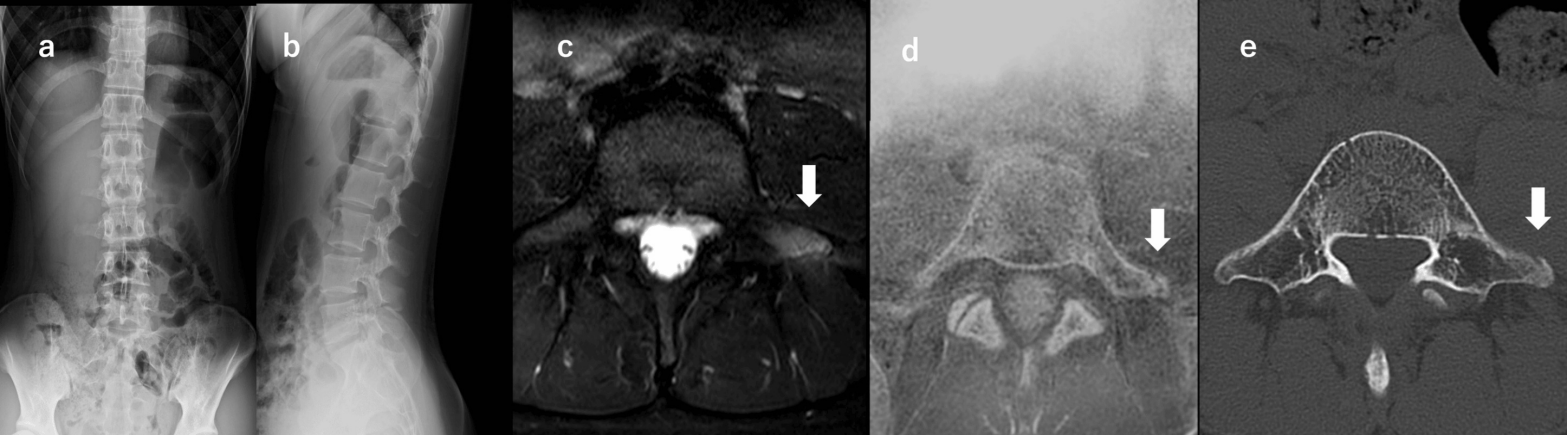
Sequential imaging findings of an L5 transverse process stress fracture in Case 2 (a, b) Plain radiographs showing no abnormalities, (c) Axial Short Tau Inversion Recovery (STIR) MRI demonstrating high signal intensity at the tip of the left L5 transverse process and adjacent soft tissue (white arrow), (d) MR bone imaging revealing a fracture line and avulsion at the tip of the left L5 transverse process (white arrow), (e) Axial CT image at four weeks confirming bone union (white arrow).

A 15-year-old female junior high school ballet dancer reported buttock pain during running three weeks before presentation, which also restricted ballet practice. She had no history of trauma, and her symptoms gradually progressed. On examination, she experienced left buttock pain during trunk flexion, extension, and rotation. The straight leg raise was 90/90 without tightness, and no neurological deficits were detected. Plain radiographs were unremarkable. MRI axial STIR sequences demonstrated signal alteration in the left L5 transverse process. MR bone imaging revealed a fracture line at the tip of the left L5 transverse process. An L5 transverse process stress fracture was diagnosed, and conservative management with activity restriction and rehabilitation was undertaken. Pain improved after about three weeks, and CT at four weeks confirmed union (Figure 2). Training was progressively resumed and return to play was achieved at eight weeks.

Table 1 compares clinical features, imaging findings, and outcomes in Case 1 and Case 2 patients.

**Table 1:** Comparison of clinical features, imaging findings, and outcomes in the two cases presented SLR: Straight leg raise.

Variable	Case 1 (Football Player, L3)	Case 2 (Ballet Dancer, L5)
Age / Sex	16 years / Male	15 years / Female
Sport	Football	Classical Ballet
Symptoms	Progressive low back pain for two months	Gradual onset of buttock pain for three weeks
History of Trauma	None	None
Physical Findings	Pain during trunk flexion, extension, and rotation; tightness on SLR (60/60)	Pain during trunk flexion, extension, and rotation; normal SLR (90/90)
Radiograph	No abnormalities	No abnormalities
MRI (STIR)	High signal at the left L3 transverse process and adjacent soft tissue	High signal at the tip of the left L5 transverse process
MR Bone Imaging	Fracture line and callus-like change	Fracture line and avulsion at the tip
CT (Follow-up)	Bone union at four weeks	Bone union at four weeks
Treatment	Activity restriction and rehabilitation (focus on flexibility and stability)	Activity restriction and rehabilitation (focus on stability)
Pain Resolution	Approximately three weeks	Approximately three weeks
Return to Sport	Eight weeks	Eight weeks

## Discussion

Low back pain represents a prevalent clinical issue among adolescent athletes, with reported prevalence rates ranging from 10% to 15% [1,2]. The prevalence differs across sports, affecting 27% of volleyball players, 15% of football players, and 11% of ballet dancers [2,11]. Lumbar transverse process fractures are generally associated with high-energy blunt trauma, such as traffic accidents or falls [3,11]. In the sports setting, these injuries have also been reported following tackles, falls, or landings [4,12]. Shahriari et al. reported that among 5,229 trauma patients, 2.2% sustained lumbar fractures, and transverse process fractures accounted for 70%, representing the most common fracture pattern [13]. In sports-related trauma, Gertzbein et al. found that approximately 29% of 119 spinal fractures caused by skiing or snowboarding involved the transverse process [5]. Tewes et al. [4] described cases of transverse process fractures in National Football League (NFL) athletes; likewise, Brynin et al. [12] reported a case of a similar fracture in a football player. These findings indicate that transverse process fractures are not uncommon in the context of both traffic- and sports-related spinal trauma. However, stress fractures of the lumbar transverse process are rare and have been only infrequently reported, with a limited number of case reports available [6-8].

The mechanism of lumbar transverse process fractures involves not only direct blunt trauma but also traction forces generated by muscular contractions [9,10]. Anatomically, the transverse processes from L1 to L4 serve as attachment sites for multiple muscles and fascial structures, creating concentrated multidirectional tensile forces. The psoas major originates from the anteromedial aspects of the L1-L5 transverse processes, exerting strong anterior traction during hip flexion and trunk stabilization. The quadratus lumborum attaches to the superior and inferior margins of the L1-L4 transverse processes, applying lateral and rotational traction during trunk movements. Barker et al. further demonstrated in anatomical studies that the middle layer of the lumbar fascia (MLF) is firmly anchored to the tips of the L2-L4 transverse processes and is continuous with the transversus abdominis (TrA) [9]. The TrA reaches the anterior abdominal wall, where it surrounds the waist like a corset and supports the lumbar vertebrae [10]. Through this connection, the MLF/TrA complex contributes to the segmental stability of the lumbar spine [9]. In cadaveric experiments, tension applied to the MLF was transmitted directly to the transverse processes, resulting in fractures [14]. Among lumbar levels, the L3 transverse process bears the largest cumulative cross-sectional area of muscular and fascial attachments, making it particularly susceptible to tensile stress and consistent with reports that L3 is the most frequent site of transverse process fractures [10]. Therefore, fractures of the L1-L4 transverse processes can be understood as primarily arising from traction stress generated by the combined action of the psoas major, quadratus lumborum, and the MLF/TrA complex.

In our case of an adolescent football player with an L3 transverse process stress fracture, there was no history of acute trauma, and the athlete experienced a gradual worsening of low back pain. We infer that decreased flexibility, combined with repeated trunk flexion and extension, rotation, and hip flexion associated with shooting and cutting maneuvers, imposed multidirectional tensile forces on the L3 transverse process, where fascial and muscular attachments are greatest, ultimately leading to a stress fracture.

The second case involved a ballet dancer with an L5 transverse process stress fracture without any history of acute trauma, and the athlete experienced a gradual worsening of buttock pain. The psoas major, as at other lumbar levels, attaches to the anteromedial aspect of the transverse process at L5. By contrast, the L5 transverse process is anatomically distinct because the iliolumbar ligament has a strong insertion at its tip, extending to the iliac crest [10]. Reis et al. reported that traumatic L5 transverse process fractures may result from traction of the iliolumbar ligament [15]. This ligament serves as a key stabilizer linking the lumbar spine and pelvis, transmitting substantial forces during trunk and pelvic movements [10]. Previous literature has reported that forward flexion of the trunk combined with posterior pelvic tilt increases tension on the iliolumbar ligament, whereas contraction of spinal stabilizing muscles such as the multifidus and erector spinae reduces this tension and contributes to spinal stability [16]. In our ballet dancer case, the repeated trunk flexion and extension required in classical ballet, particularly during movements that emphasize spinal stability, likely generated repetitive stress on the iliolumbar ligament. Furthermore, it is conceivable that insufficient activation of spinal stabilizing muscles, such as the multifidus and erector spinae, led to compensatory loading through the iliolumbar ligament, thereby producing repetitive stress and ultimately resulting in a traction stress fracture at the tip of the L5 transverse process, where the iliolumbar ligament attaches.

Taken together, these two cases illustrate that stress fractures of the lumbar transverse processes occur through different mechanisms depending on the vertebral level: at L3, through the cumulative traction of the psoas, quadratus lumborum, and the MLF/TrA complex; and at L5, through the unique involvement of the iliolumbar ligament in addition to the muscular attachment of the psoas major.

Transverse process fractures are often challenging to detect on plain radiographs. Overlap with the ilium, the iliopsoas muscle, and intestinal gas reduces visibility on anteroposterior views, and minor displacements are easily missed. As a result, these fractures are frequently overlooked due to their low diagnostic priority, limited recognition, and nonspecific clinical presentation [12,17]. Stress fractures typically require time for radiographic changes, such as fracture lines or sclerosis, to become apparent, contributing to delayed diagnosis. MRI is valuable in this setting, as it permits early identification of bone marrow edema and periosteal soft tissue changes. In lumbar stress fractures such as spondylolysis, fat-suppressed sequences (e.g., STIR) enable early detection of signal changes in the pars interarticularis, and the importance of early recognition for bone union has been reported [18]. Accordingly, MRI is generally performed first when a stress fracture is suspected, with CT reserved for fracture line evaluation.

McGuire et al. reported a transverse process stress fracture that was undetectable on radiographs but confirmed on MRI [6]. Conversely, Bali et al. described a professional cricket player whose chronic low back pain was attributable to a nonunion of a transverse process stress fracture, with displacement visible on radiographs and multilevel involvement confirmed by CT [7]. These observations emphasize that delayed diagnosis can contribute to chronic pain and highlight the clinical importance of early detection with MRI and CT. In adolescent athletes, however, CT carries the drawback of radiation exposure. Recently, advances in MRI technology have enabled visualization of cortical bone using MR bone imaging. This technique is based on a three-dimensional radiofrequency (RF)-spoiled steady-state gradient-echo (RSSG) sequence, which captures very short T2 components that are not visible on conventional MRI, thereby providing a CT-like depiction of cortical bone while avoiding radiation exposure.

Okuyama et al. demonstrated that MR bone imaging achieves almost perfect inter-rater (κ = 0.92) and inter-modality (κ = 0.83-0.84) agreement with CT in the evaluation of adolescent lumbar spondylolysis, confirming its reliability for cortical bone assessment comparable to CT. These findings support the validity of MR bone imaging as a noninvasive diagnostic tool for evaluating stress fractures involving cortical bone, especially in young athletes, where minimizing radiation exposure is important [19].

In our series, plain radiographs failed to demonstrate abnormalities in both cases, leading to an MRI with MR bone imaging. In the first case, fat-suppressed MRI revealed signal changes in the left L3 transverse process and adjacent soft tissues, and MR bone imaging demonstrated a fracture line with partial callus-like changes. These findings initially suggested a tendency toward healing, yet the resumption of physical activity was likely responsible for progression to complete fracture with subsequent pain. In the second case, fat-suppressed MRI revealed signal changes in the left L5 transverse process, with MR bone imaging confirming an avulsion at the tip. Follow-up CT after symptom resolution demonstrated bone union in both patients. Because MR bone imaging successfully identified the fracture lines, CT was required only once, at the time of union confirmation. MR bone imaging may serve as a useful alternative to CT in the evaluation of stress fractures, particularly in adolescent athletes.

Transverse process fractures represent stable injuries that generally respond well to conservative treatment [3,20]. Kaya reported that routine corset application is not obligatory [20]. Although reports on long-term outcomes remain limited, the majority indicate minimal or absent residual disability [3]. In traumatic cases, Brynin and Gardiner [12] documented a return-to-play time of about four weeks in a high school football athlete, while Tewes et al. [4] observed a mean recovery period of 3.5 weeks across 29 NFL players. Conversely, McGuire et al. reported that rowing athletes with stress fractures initiated gradual training at seven weeks and achieved full return by 13 weeks [6]. Unlike acute trauma, stress fractures result from repetitive mechanical loading, predisposing athletes to recurrent pain at the fracture site despite transient improvement. Because the lumbar spine experiences high mechanical stress during physical activity, rehabilitation is important for preventing recurrence. Rehabilitation strategies should emphasize avoiding pain-provoking activities, early recovery of flexibility, and preservation of spinal and core stabilizer strength (including transversus abdominis, multifidus, and erector spinae), with progressive transition from low-impact aerobic exercise to competitive return.

In the present cases, rehabilitation was initiated immediately after diagnosis. In Case 1, the program focused on gaining flexibility and enhancing core and spinal stability, whereas in Case 2, the main emphasis was on core and spinal stability. Both athletes experienced pain resolution within approximately three weeks, and CT imaging at four weeks confirmed bone healing. Training intensity was gradually increased, enabling both athletes to return to sport around eight weeks.

Although the overall prognosis of transverse process fractures is generally favorable, delayed recognition in non-traumatic cases has been associated with nonunion and chronic low back pain [7,8]. Therefore, early diagnosis combined with conservative treatment is essential to ensure a safe and effective return to sport in adolescent athletes. Because lumbar transverse process stress fractures lack specific clinical features and are often underrecognized or misdiagnosed as nonspecific low back pain, they should be considered in the differential diagnosis of adolescent athletes presenting with low back pain.

## Conclusions

We reported two rare cases of lumbar transverse process stress fractures, which may be easily overlooked when no history of acute trauma is present. Early diagnosis with MRI, including MR bone imaging, enabled timely recognition and effective conservative management in both patients, allowing safe return to sport at approximately eight weeks. These cases emphasize the importance of considering lumbar transverse process stress fractures in the differential diagnosis of low back pain in adolescent athletes and underscore the value of prompt imaging evaluation to ensure favorable outcomes and prevent chronic disability. Clinicians should maintain a high index of suspicion for lumbar transverse process stress fractures in adolescent athletes with persistent low back pain, even in the absence of trauma.
